# A novel method for the quantification of fatty infiltration in skeletal muscle

**DOI:** 10.1186/s13395-016-0118-2

**Published:** 2017-01-10

**Authors:** Nicole K. Biltz, Gretchen A. Meyer

**Affiliations:** 1 Program in Physical Therapy, Washington University in St. Louis, 4444 Forest Park Blvd, St. Louis, 63108 MO USA; 2 Departments of Neurology, Biomedical Engineering and Orthopaedic Surgery, Washington University, St. Louis, MO USA

**Keywords:** Fatty infiltration, Intramuscular fat, IMAT, Muscle lipid

## Abstract

**Background:**

Fatty infiltration of the skeletal muscle is a common but poorly understood feature of many myopathies. It is best described in human muscle, where non-invasive imaging techniques and representative histology have been optimized to view and quantify infiltrating fat. However, human studies are limited in their ability to identify cellular and molecular mechanisms regulating fatty infiltration, a likely prerequisite to developing targeted interventions. As mechanistic investigations move to small animals, studies may benefit from new or adapted imaging tools optimized for high resolution and whole muscle quantification.

**Results:**

Here, we describe a novel method to evaluate fatty infiltration, developed for use with mouse muscle. In this methodology, muscle cellular membranes and proteins are removed via decellularization, but fatty infiltrate lipid is spared, trapped in its native distribution in a transparent extracellular matrix construct. This lipid can then be stained with visible or fluorescent dyes and imaged. We present three methods to stain and evaluate lipid in decellularized muscles which can be used individually or combined: (1) qualitative visualization of the amount and 3D spatial distribution of fatty infiltration using visible lipid soluble dye Oil Red O (ORO), (2) quantitative analysis of individual lipid droplet metrics (e.g., volume) via confocal imaging of fluorescent lipid soluble dye boron-dipyrromethene (BODIPY), and (3) quantitative analysis of total lipid content by optical density reading of extracted stained lipid.

This methodology was validated by comparing glycerol-induced fatty infiltration between two commonly used mouse strains: 129S1/SvlmJ (129S1) and C57BL/6J (BL/6J). All three methods were able to detect a significant increase in fatty infiltrate volume in the 129S1 muscle compared with that in BL/6J, and methods 1 and 2 additionally described a difference in the distribution of fatty infiltrate, indicating susceptibility to glycerol-induced fatty infiltration is strain-specific.

**Conclusions:**

With more mechanistic studies of fatty infiltration moving to small animal models, having an alternative to expensive non-invasive imaging techniques and selective representative histology will be beneficial. In this work, we present a method that can quantify both individual adipocyte lipids and whole muscle total fatty infiltrate lipid.

**Electronic supplementary material:**

The online version of this article (doi:10.1186/s13395-016-0118-2) contains supplementary material, which is available to authorized users.

## Background

The accumulation of adipocytes within the skeletal muscle is a common manifestation of muscle pathology, documented in at least ten primary and secondary myopathies [1–11]. Over the past two decades, this phenomenon has received increasing focus as it is strongly correlated with insulin resistance [2, 6, 12–15] and/or poor functional capacity [16–20] across a number of disease states and populations, from young boys with Duchenne muscular dystrophy to aging adults with type 2 diabetes. However, despite its prevalence and association with dysfunction, little is known about the signaling mechanisms that regulate this process of fatty infiltration, and considerable debate continues about its origin and consequences (reviewed in [21]). This is partly due to the fact that the vast majority of studies in this area have been in humans, where causation is difficult to demonstrate. Genetic manipulation in small animals, mice in particular, can be a powerful tool for defining the mechanisms behind cellular and tissue functional regulation. However, the size scale of small animal models frequently allows and demands different experimental methodology than what is developed for human studies.

The goal of this work is to provide a new measurement tool for quantifying fatty infiltration in small animal models.

Clinically, and in human research studies, fatty infiltration is most frequently assessed via non-invasive imaging modalities such as computed tomography (CT), magnetic resonance imaging (MRI), and ultrasound (e.g., [9, 22–24]). This typically involves acquisition of image slices of the muscle cross-section within a defined region of interest (e.g., quadriceps at mid-thigh or supraspinatus at the distal connection of the scapular spine) that are then either qualitatively graded (e.g., [9]) or quantified via pixel intensity-based thresholding (e.g., [24]). The major challenges for translating these methods to animal models are cost and availability of sufficiently high-powered machines to provide anatomical resolution in small animals, though a few studies have used CT or MRI to quantify fatty infiltration in mice and sheep [25, 26]. Another challenge with these methods is that standard machines lack the spatial resolution to distinguish between adipocytes and muscle fibers [27, 28]. Thus, areas that are identified as “fat” or “muscle” via thresholding may, in actuality, be mixtures of adipocytes and muscle fibers, leading to errors in quantification.

Because of these limitations, when fatty infiltration is assessed in small animal models, it is most frequently evaluated via histology (e.g., [29–32]). Compared with CT and MRI, histology is relatively inexpensive and allows the visualization of individual adipocytes with standard staining techniques such as Oil Red O, hematoxylin and eosin (H&E), and immunohistochemistry. However, histological sections are frequently only qualitatively evaluated (e.g., [31]) or scored on a rating scale to quantify differences between treatment groups (e.g., [29]) and, like CT and MRI techniques, are sampled from only one or a few places within the muscle volume (e.g., [32]). This limited sampling provides an incomplete picture of fatty infiltration in muscle as accumulation of adipocytes can be very spatially heterogeneous across the muscle volume [33]. Biochemical extraction of triglyceride from the skeletal muscle has been used extensively as a comprehensive and inexpensive quantification technique to study dynamic changes in intramyocellular lipid stores (e.g., [34]). However, in intact muscle with fatty infiltration, this technique is unable to distinguish between intramyocellular and extramyocellular stores of lipid [35]. Thus, currently available methodologies for quantifying fat in muscle are limited either by cost, sampling, or specificity for fatty infiltrate.

In this work, we describe a novel method for assessing fatty infiltration that can be used for high-resolution imaging and comprehensive quantification. This method is based on a validated decellularization technique that removes the majority of cellular structures, but retains the large lipid droplets present in fatty infiltrate adipocytes [36]. We demonstrate the ability of this method to (1) provide a qualitative visualization of the pattern of fatty infiltration through a muscle volume, (2) quantify individual adipocyte metrics and 3D patterning, and (3) provide a quick and inexpensive quantification of fatty infiltrate lipid volume. We then use these measures to compare experimentally induced fatty infiltration between two commonly used mouse strains, 129S1/SvlmJ and C57BL/6J, and document substantial inter-strain differences not only in the total volume of fatty infiltration but also in spatial distribution patterns. These differences suggest that the background strain of mouse may contribute significantly to the susceptibility of different disease models to fatty infiltration.

## Methods

### Experimental design

Experiments were performed on male 129S1/SvlmJ (129S1; *n* = 14) and C57BL/6J (BL/6J; *n* = 7) mice at 2–3 months of age (The Jackson Laboratory). Unless otherwise noted, all figures use muscles from the 129S1 mice. Fatty infiltration of muscles was induced by injecting an anesthetized mouse with 10 μL of a sterile 50% *v*/*v* glycerol (GLY) solution in PBS into the midbelly of either the 5th toe extensor digitorum longus (EDL) muscle or tibialis anterior (TA) muscle (when additional sample volume was required), similar to methods previously described [37]. Injection of 10 μL sterile saline (SAL) was similarly delivered to control muscles. Injections into the TA muscles were delivered through the skin, while injections into the 5th toe EDL muscles were delivered through a small subcutaneous incision at the medial aspect of the ankle that provided access to the distal portion of the EDL. Injections were delivered via 29-gauge needle inserted along the longitudinal muscle dimension. Following injection, the incisions were closed and the mice were allowed to recover for 3 weeks at which point mice were euthanized via cervical dislocation and the muscles were collected. All procedures were performed in accordance with the National Institutes of Health’s Guide for the Use and Care of Laboratory Animals and were approved by the Animal Studies Committee of the Washington University School of Medicine.

### Muscle decellularization

**Fig. 1:**
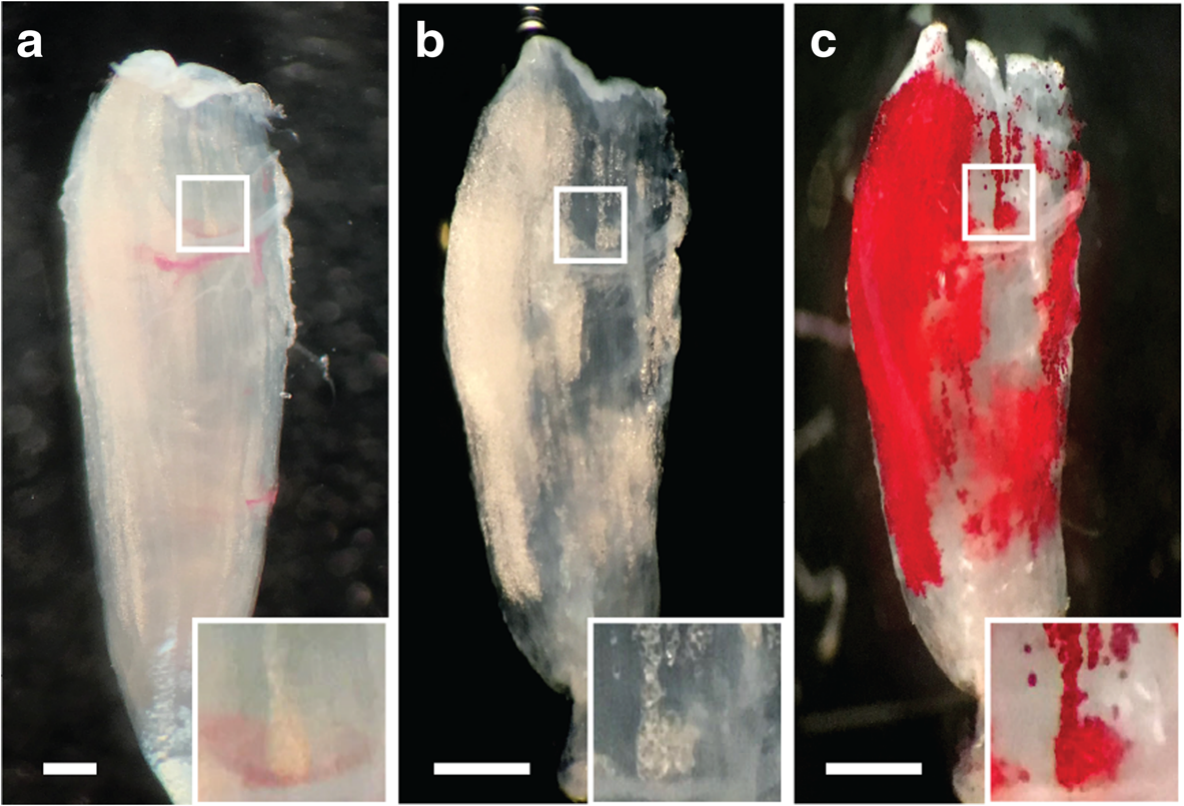
Illustration of qualitative inspection of fatty infiltration by decellularization and oil red O (ORO) staining. **a** A representative isolated 5th toe EDL muscle. **b** The same muscle following decellularization. Decellularization removes myocellular proteins but spares large lipid droplets visible as spherical structure with increased reflectance in a semi-transparent construct. **c** The same muscle following staining with the lipid soluble dye ORO where lipid droplets are stained *bright red*. Some superficial lipid is visible in the intact muscle and can be tracked through the decellularization and staining process (*inset*, using the proximal vessel as an anatomical landmark). *Scale bars* are 500 μm

Following dissection, muscles were decellularized in a 1% solution of sodium dodecyl sulfate (SDS, Sigma Aldrich) in PBS with agitation, similar to methods previously described [36]. This treatment removes myocellular components but spares the large lipid droplets of fatty infiltrate adipocytes which remain trapped within the extracellular matrix (ECM) (Fig. 1a, b). Fresh SDS was applied daily, and the muscles were removed from solution when fully transparent: 24 h for the 5th toe EDL and 3 days for the TA. The muscles were then washed three times in PBS and fixed in 3.7% formaldehyde for 48 h.

### Visualization and quantification of lipid with Oil Red O

To enhance visualization of retained lipid, the decellularized muscles were incubated with Oil Red O (ORO, Sigma Aldrich). ORO was dissolved in a 60% isopropanol solution and added to decellularized muscles for 45 min with agitation. Muscles were then washed overnight with 1% SDS to remove unbound ORO, yielding bright red droplets in a transparent ECM construct (Fig. 1c). Whole muscle images were taken through a dissecting microscope for a qualitative evaluation of the distribution of fatty infiltration. The muscles were then placed in 200 μL isopropanol with agitation to extract the stained lipid. ORO concentration was read from 75 μL samples in a 96-well plate by Synergy II multi-mode plate reader (Bio-Tek) in duplicate at 500 nm.

### Visualization and quantification of lipid with fluorescent imaging

**Fig. 2:**
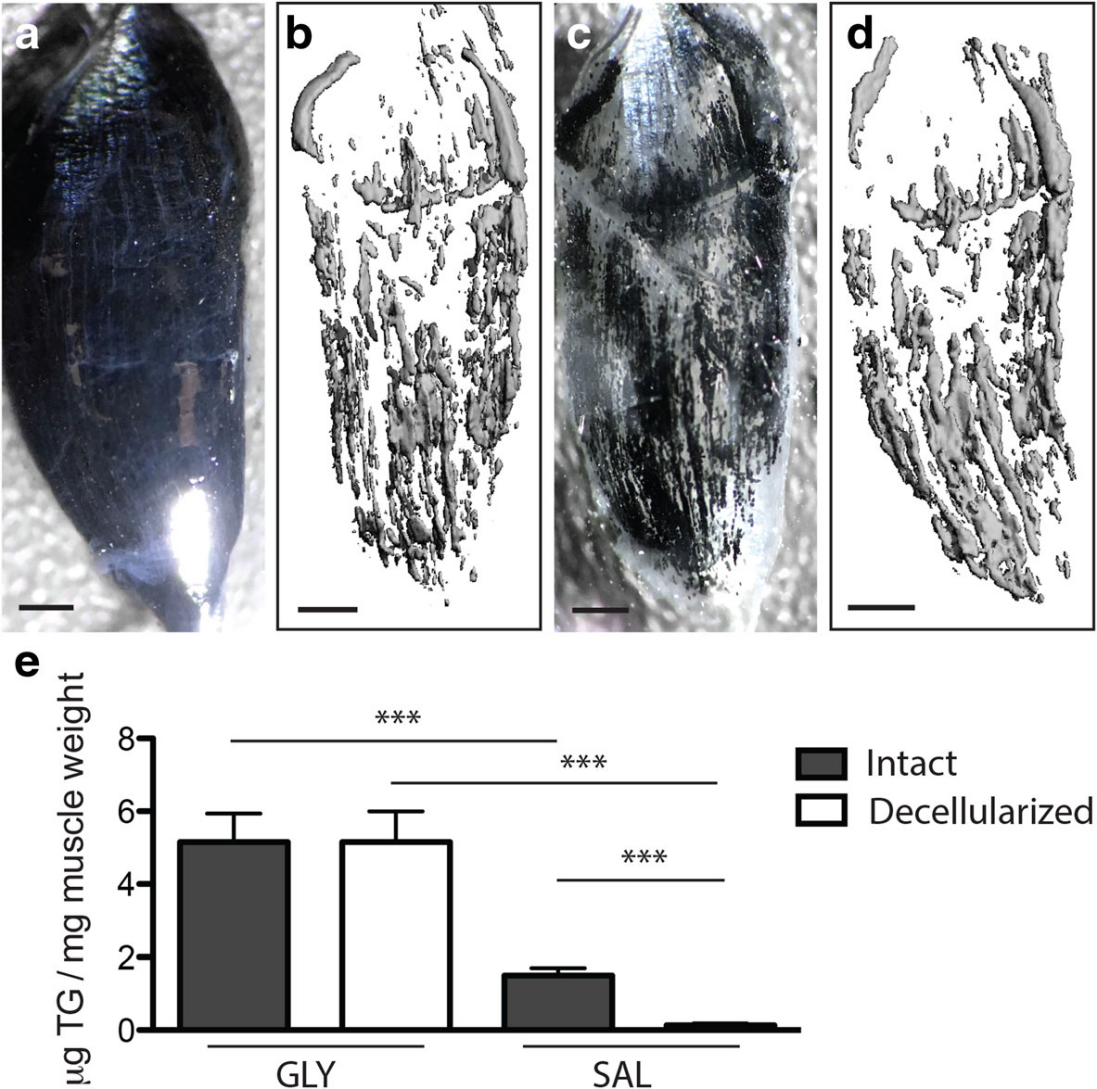
Fatty infiltrate lipid, but not intramyocellular lipid, is retained in decellularized muscles. **a** An isolated 5th toe EDL muscle stained with osmium tetroxide. The opaque black appearance of the muscle is due to osmium binding with intramyocellular lipid and phospholipids in fiber membranes in addition to fatty infiltrate lipid. **b** 3D rendering of the osmium signal acquired via μCT. Thresholding was used to isolate the high signal intensity originating in fatty infiltrate from the low signal originating from myocellular lipids. **c** Decellularization of the same muscle following imaging. Retained lipid, stained black, has the same qualitative distribution pattern as the 3D μCT rendering. **d** Re-imaging of the decellularized muscle yields a similar 3D μCT rendering to that obtained from the intact muscle. Note that the shift in orientation in the lower quadrant is the result of bending during re-embedding in agarose. **e** Triglyeride content in tibialis anterior (TA) muscles quantified by lipid extraction and normalized to pre-decellularized muscle weight. Both intact and decellularized muscles treated with GLY have significantly higher triglyceride content than intact and decellularized muscles treated with SAL, respectively. *Scale bars* are 500 μm. In the SAL treatment group, nearly all lipids present in the intact group is eliminated in the decellularized group. ***p < 0.0005

To quantify lipid droplet metrics, the decellularized muscles were incubated with the fluorescent lipid soluble dye BODIPY (1:200 BODIPY 493/503 (Life Technologies) in PBS) for 20 min. BODIPY signal distribution was imaged using an Olympus FV1200 scanning confocal microscope. Quantification was performed by acquiring stacks with 10-μm step size over the entire muscle volume. Individual BODIPY-positive areas were visualized and measured using the ImageJ (NIH) software in a semi-automated process (Additional file 1: Figure S2). First, areas of high signal intensity were thresholded in each image of the stack using the Intermodes automatic thresholding algorithm. This algorithm was chosen because it minimized registration of out-of-plane fluorescence. Then, images were segmented using the Watershed algorithm and regions of interest registered using the Analyze Particles algorithm. These three algorithms are core functions of ImageJ, and this automatic registration procedure is similar to that frequently used for quantifying DAPI stained nuclei [38]. Any remaining unregistered lipid droplets were identified by hand by fitting a circular or elliptical region of interest (ROI) at the slice of maximum signal intensity to minimize out-of-plane fluorescent artifacts. Post-processing of ROIs was performed using a Matlab algorithm to eliminate repeat registration of lipid droplets that spanned multiple slices. Finally, the area of each ROI was converted to a sphere or ellipsoid volume using the minor axis distance as the *Z*-plane radius. The centroid of the volume was recorded as the *X*-*Y* centroid of the ROI and the *Z*-position as the slice of maximum signal intensity. This semi-automated registration was validated against manual registration (hand fitting ROIs) by two independent researchers. The intraclass correlation coefficients for total lipid volume were above 0.95 between the semi-automated and manual registration for both researchers. Visualization of the 3D map was performed with Matlab and qualitatively compared with ORO staining completed post-confocal imaging (Fig. 2). Adipocyte metrics were also computed in Matlab. Total lipid volume was the summation of individual ROI volumes, total number of adipocytes was computed as the number of ROIs, and average adipocyte volume was the average volume of ROIs. The nearest neighbor index is a measure of spatial clustering; the smaller the nearest neighbor index, the more the pattern exhibits clustering. It is computed as the average nearest neighbor Euclidian distance for each ROI divided by the average nearest neighbor distance in a hypothetical random distribution.

### Visualization and quantification of lipid with micro-computed tomography

To image lipid distribution in intact muscle, freshly isolated muscles were stained with a 1% solution of osmium tetroxide (Polysciences Inc.) in PBS for 30 min. The muscles were then washed overnight in PBS, embedded in 1% agarose and scanned at 10 μm voxel resolution using a Scanco μCT 40 (Scanco Medical, Zurich, Switzerland). Images were generated by applying a threshold of 400 (on a 0–1000 scale) to the tissue. Following initial image acquisition, the muscles were removed from agarose, decellularized as described above and re-imaged with identical scan and threshold parameters.

### Histological evaluation of fatty infiltration

Muscles selected for histological analysis were dissected, embedded in the gastrocnemius (gastroc) muscle, and flash frozen in liquid nitrogen-cooled isopentane. Embedding the 5th toe EDL in the muscle was found to be superior to embedding in other compounds, such as OCT, in preservation of tissue morphology and minimization of edge artifacts due to freezing and sectioning. The gastroc muscle was obtained from GFP reporter mice (C57BL/6-Tg (CAG-EGFP), Jackson Labs) that were experimental animals in another, unrelated experiment. The soleus muscle was dissected free from the gastroc and the EDL was laid in its place. Then the medial and lateral heads of the gastroc were wrapped around the EDL and secured together with suture glue creating a muscle composite where the GFP signal allowed precise identification of the EDL boundary.

Composites were cut into 10 μm sections on a cryostat (Leica Biosystems) at −20 °C. Serial sections were obtained through two 110-μm-thick sections of the muscle, one proximal and one distal. These were sequentially immunostained with perilipin (1:400, Progen Biotechnik) to visualize lipid boundaries and stained with hematoxylin and eosin (H&E) to visualize tissue morphology. Images were acquired of the entire muscle cross-section of each serial section, and adipocyte volumes were computed similarly to confocal quantification using manually drawn ROIs around adipocytes. Some additional sections were also stained with ORO prior to immunostaining to visualize lipid. These were mounted on pre-chilled slides and vapor fixed overnight in 37% formaldehyde. ORO was mixed and applied as described for decellularized muscles but was left on sections for only 5 min with continual agitation. Sections were then gently washed and imaged, and stained lipid was extracted with isopropanol.

### Triglyceride extraction and analysis

Total triglyceride, which includes the contents of lipid membranes, intramyocellular lipid droplets, and fatty infiltrate lipid, was determined in the TA muscles rather than in the 5th toe EDL due to the need for larger tissue samples. Total triglyceride was determined by homogenizing either fresh or decellularized muscles in a chloroform-methanol (2:1 *v*/*v*) mixture, followed by centrifugation at 4 °C. The organic phase was then taken to dryness and resuspended in Triglycerides Reagent (Thermo Fisher Scientific). Triglyceride was quantified via absorbance readings of the suspension according to the manufacturer’s protocol and normalized to fresh muscle weight (measured prior to decellularization).

### Computation and statistical analysis

For comparisons between the intact and decellularized muscles and between the 129S1 and BL/6J strains, a two-tailed Student’s *t* test was used with significance set at *p* < 0.05. For comparisons with two treatment groups (decellularization and GLY), a two-way ANOVA was run with significance set at *p* < 0.05 and a Tukey post hoc test. For comparisons between confocal and ORO extraction measurements of lipid volume, a Pearson’s correlation was run. All analyses were conducted using PRISM (Graphpad).

## Results

The majority of analyses presented in this manuscript utilize the 5th toe EDL muscle due to its small size and extensive utilization in physiology studies. However, decellularization is equally effective for visualizing fatty infiltrate lipid in other muscles and muscle groups maintained in their anatomical position attached to the skeleton (Additional file 2: Figure S1).

### Qualitative visualization of the pattern of fatty infiltration

Decellularization of GLY-injected 5th toe EDL muscles yielded a semi-transparent construct containing a number of spherical structures of increased reflectance (Fig. 1a, b). These structures stained brightly with the lipid soluble dye ORO indicating that they were lipid retained within the decellularized muscle (red in Fig. 1c). Some of these lipid structures could be visualized superficially in the intact EDL prior to decellularization (Fig. 1 inset), suggesting they were not an artifact of the decellularization protocol but represented existing lipid stores within the muscle.

To confirm that decellularization treatment retained lipid structures in their native distribution, the intact muscles were stained with osmium tetroxide and imaged with μCT to visualize intramuscular lipid (Fig. 2a, b). Following imaging, the muscles were decellularized and the retained osmium-stained lipid inspected visually (black in Fig. 2c) and re-imaged with μCT (Fig. 2d). The spatial distribution of lipid in the intact muscles was nearly identical to the spatial distribution of lipid following decellularization indicating that lipids retained in the decellularized muscle are existing structures from the intact muscle in their native distribution (compare Fig. 2b with d). Furthermore, few if any osmium-stained structures appeared to be lost with decellularization. To further explore lipid retention in decellularization, triglycerides were quantified in GLY- and SAL-injected tibialis anterior (TA) muscles via lipid extraction. GLY-injected intact muscles had significantly greater triglycerides than SAL-injected (Fig. 2e), as expected, since GLY is a stimulus for fatty infiltration [37] and mouse TA muscles contain little to no extramyocellular fat in absence of stimulus [39]. There was no difference in triglyceride content between the intact and decellularized GLY-injected TA muscles, again indicating that the majority of fatty infiltrate lipid was retained by decellularization (Fig. 2e, GLY). However, there was a significant reduction in triglyceride content, almost to zero, in decellularized SAL-injected TA muscles (Fig. 2e, SAL). This suggests that the decellularization protocol selectively retains fatty infiltrate lipid while removing intramyocellular lipid, effectively separating these two intramuscular lipid stores.

### Quantitative imaging of fatty infiltrate lipid droplets

**Fig. 3:**
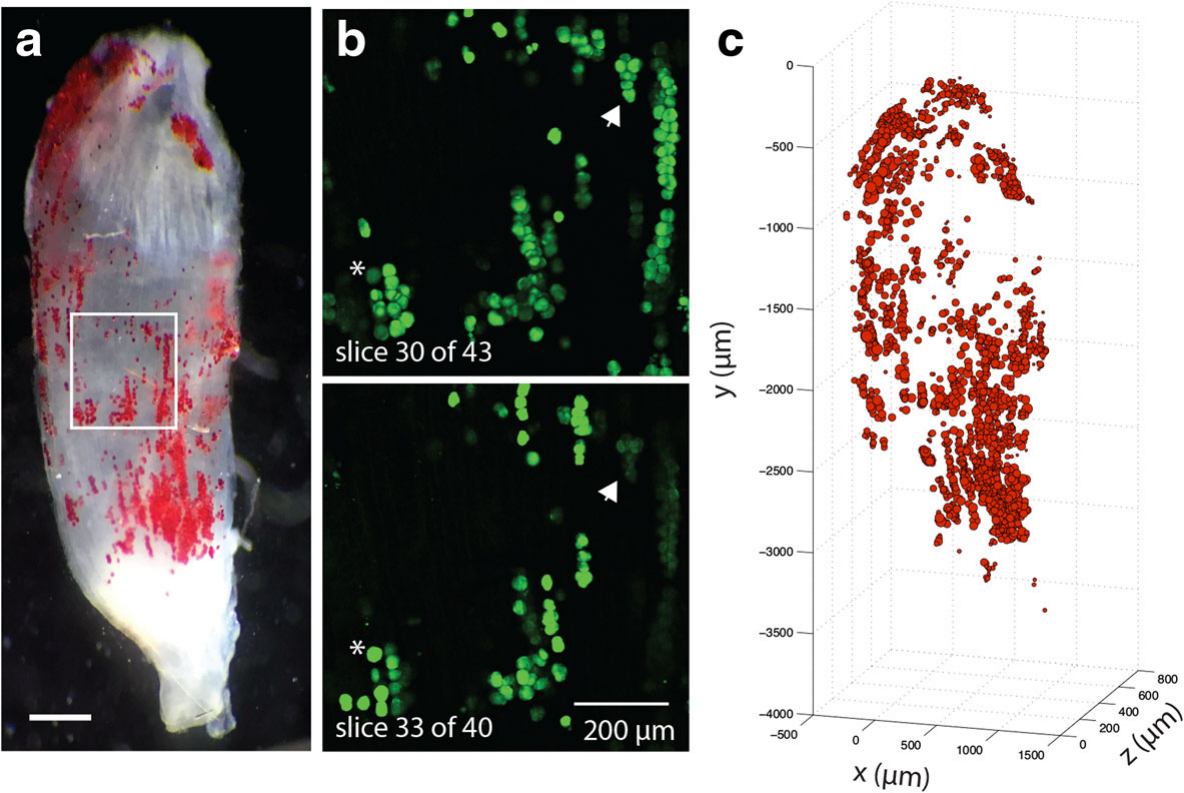
Illustration of quantitative analysis of lipid droplet metrics by fluorescent confocal microscopy. **a** ORO (*red*)-stained decellularized 5th toe EDL muscle used for qualitative reference. *Scale bar* is 500 μm. **b** Two representative image slices from a confocal stack acquired of the image area boxed in panel **a**. BODIPY-positive (*green*) circular areas can be clearly differentiated. Some of the same areas can be seen in both slices (*asterisks* and *arrows*) but have maximal signal strength only in one (the slice where the ROI was registered). **c** The 3D reconstruction performed on all registered ROIs. Qualitative comparison of this map and the ORO-stained image in panel **a** shows good representation through the muscle volume

Confocal microscopic imaging of a lipid soluble fluorescent dye, BODIPY, was used to delve further into quantitative metrics related to fatty infiltration such as number of infiltrating adipocytes, average adipocyte volume, total volume of fatty infiltrate, and spatial clustering between adipocytes in decellularized muscles. In a thin muscle such as the 5th toe EDL, a ×10 objective can distinguish individual lipid droplets in confocal stacks taken through the full thickness of the muscle (Fig. 3a, b). These droplets are predominately circular in the image plane making semi-automated image processing by watershed segmentation highly effective (see Additional file 3 and Additional file 1: Figure S2). Once the 3D coordinates and volume of each adipocyte are registered, quantitative metrics can be calculated, and a 3D map of fatty infiltration can be generated (Fig. 3c).

**Fig. 4:**
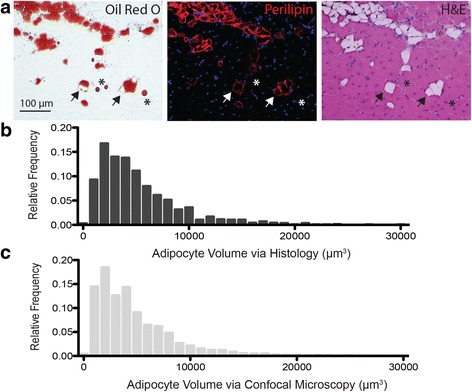
Estimated adipocyte volumes are similar between intact and decelluarized 5th toe EDL muscles. **a** Representative sequential histology demonstrating lipid withdrawal (*arrows*) and fractionation (*asterisks*) artifacts incurred by sectioning and ORO staining. Perilipin immunofluorescent signal (*red*, *center*) marks where lipid membranes remain fixed and eosin staining (*pink*, *right*) shows fiber material where ORO staining marked lipid. **b** Histogram of adipocyte volumes estimated from sequential perilipin and H&E staining of serial sections of intact muscle (combined data from four sampled muscle volumes). **c** Histogram of adipocyte volumes estimated from fluorescent confocal imaging of decellularized muscles (combined data from four muscles)

Ideally, the lipid droplet volumes computed from this method could be used as a surrogate for fatty infiltrate adipocyte volume. However, the possibility exists that lipid could fractionate or coalesce during the decellularization process leading to an under- or over-estimation of adipocyte size. To explore this possibility, two GLY-injected 5th toe EDL muscles were serially sectioned at two locations and adipocyte volume quantified via histology. ORO staining was determined to be a poor measure of adipocyte volume as even careful treatment of the section still resulted in withdrawal and fractionation of lipid from the cellular boundary, determined by sequential staining with perilipin (lipid membrane) and H&E (muscle fibers) (Fig. 4a, arrows and asterisks). Thus, quantification was performed using sequential staining for perilipin and H&E only to identify non-fiber areas bordered by perilipin. These areas were converted to volumes by tracking the same area over multiple sections (see Methods). The histogram of adipocyte volumes obtained from the intact muscles by histology (Fig. 4b) was very similar to that obtained from the decellularized muscles by confocal microscopy (Fig. 4c) suggesting that lipid droplet size measured in decellularized muscles is a good approximation of fatty infiltrate adipocyte volume.

### Qualitative and quantitative comparisons of fatty infiltration in 129S1 and BL/6J mice

**Fig. 5:**
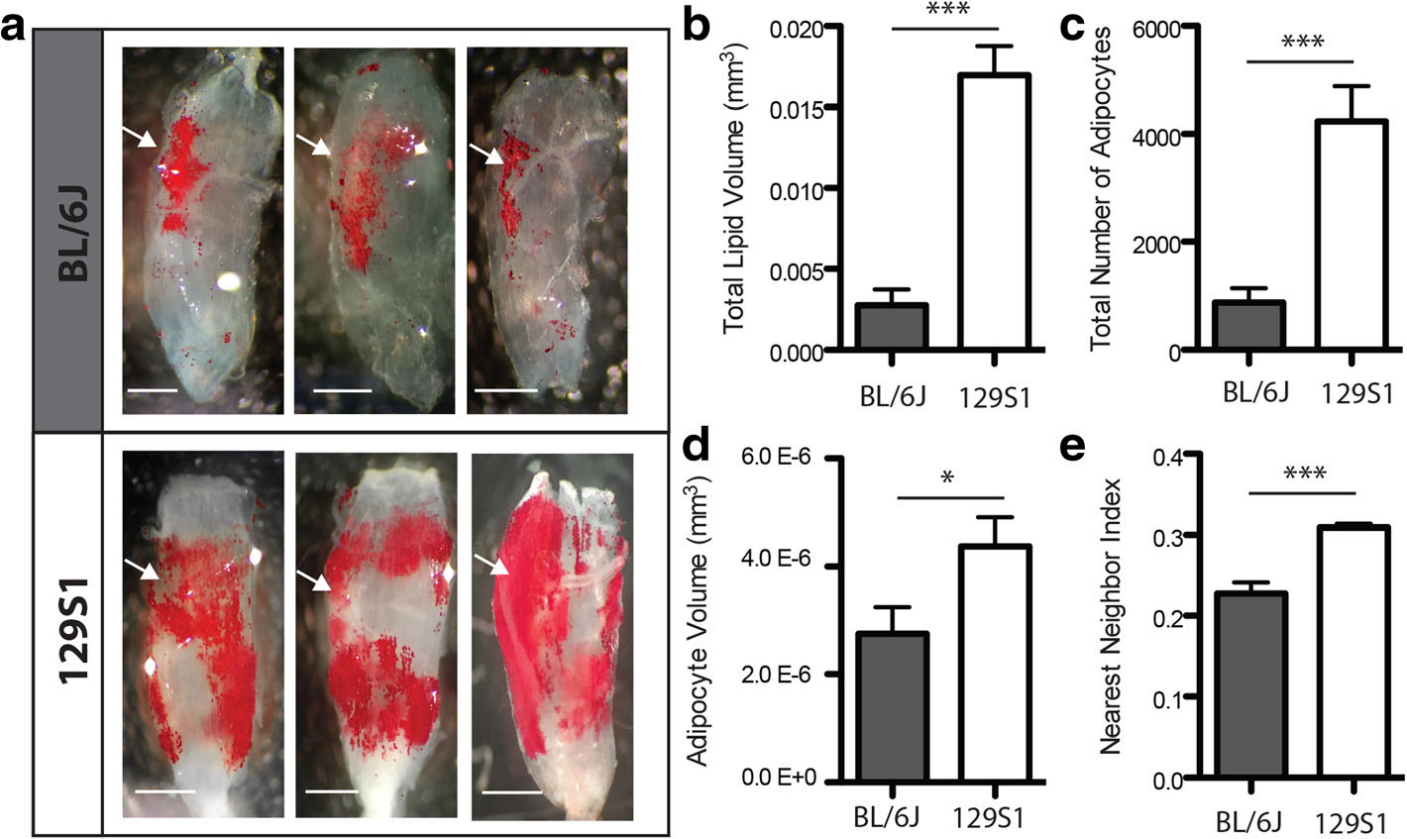
Fatty infiltration resulting from GLY injection is higher in the 129S1/Sv1mJ (129S1) mouse strain than in the C57BL/6J (BL/6J) mouse strain. **a** Images of ORO staining in decellularized muscles following GLY injection for three representative BL/6J muscles (*upper panel*) and three representative 129S1 muscles (*lower panel*). The location of the proximal vessel structure is indicated by the *arrows*. Global differences in both quantity and distribution of fatty infiltration are apparent between strains. Quantification of fatty infiltration metrics finds that **b** total lipid volume, **c** total number of adipocytes, **d** average adipocyte volume, **e** and the nearest neighbor index (a measure of spatial clustering) are significantly increased in the 129S1 muscle compared in the BL/6J. ***p < 0.0005

To demonstrate the utility of this methodology for experimental comparison of muscle fatty infiltration between conditions or groups, we have compared GLY-induced fatty infiltration between two commonly used background strains of mice: 129S1/SvlmJ (the strain used in Figs. 1, 2, 3, and 4) and C57BL/6J. These strains are thought to have intrinsic differences in their susceptibility to fatty infiltration, but quantitative comparison via histology has yielded conflicting results potentially due to sampling issues [40]. In the 5th toe EDL muscles, GLY injection caused significantly greater fatty infiltration in 129S1 than in the BL/6J muscles, evident qualitatively by inspection of ORO-stained muscles (Fig. 5a) and quantitatively by analysis of adipocyte metrics obtained via confocal analysis (Fig. 5b–e). 129S1 muscle had significantly increased total lipid volume (Fig. 5b), which was due primarily to an increase in adipocyte number (Fig. 5c) with a small, but still significant, increase in average adipocyte volume (Fig. 5d). Differences in total lipid volume and adipocyte number between strains remained significant when normalized to muscle volume, indicating that both the absolute and relative amount of fatty infiltration are significantly different between 129S1 and BL/6J muscle. Beyond the overall increased quantity of fatty infiltration in the 129S1 muscles compared with that in BL/6J, there was a striking difference in the distribution pattern. In the BL/6J muscle, fatty infiltration was predominantly clustered at the proximal vessel structure (arrows in Fig. 5a), while in the 129S1 muscle, fatty infiltration was more widespread, typically spanning the majority of the muscle volume. This distribution difference was quantified using a nearest neighbor index, a measure of spatial clustering. The 129S1 muscle had a significantly elevated nearest neighbor index, indicating a more dispersed pattern of fatty infiltration (Fig. 5e).

**Fig. 6:**
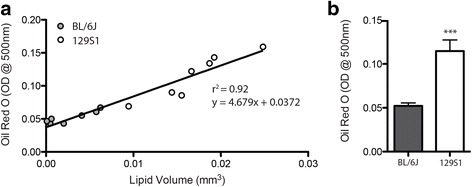
Quantification of lipid volume by optical density (OD) measurement of ORO content in extracted lipid. **a** Regression of ORO extraction vs. lipid volume measurements via fluorescent confocal microscopic analysis (data from Fig. 5). ORO OD readings are significantly correlated (*p* < 0.0001) with lipid volume measurements made prior to ORO staining. Inset shows a magnified view of the low lipid volume data points. **b** ORO OD readings are sensitive enough to detect a significant difference between strains. ***p < 0.0005

Because confocal microscopic analysis can be expensive and is intractable for large muscles, we sought to determine whether total lipid volume could be estimated via optical density (OD) measurement of extracted ORO stained lipid. OD measurements of lipid extracted following confocal microscopy are highly correlated (r^2^ = 0.92) with lipid volume calculated via image analysis (Fig. 6a). Statistical comparison of OD readings between strains demonstrates a significant increase in ORO, and by extension lipid volume, in the 129S1 muscles (Fig. 6b). Taken together, these data indicate that ORO staining of decellularized muscle can provide a quick, relatively inexpensive, and comprehensive qualitative and quantitative measure of fatty infiltration volume and distribution in muscle.

## Discussion

Current methods for quantification of muscle fatty infiltration are limited by either insufficient spatial resolution to distinguish adipocytes from muscle fibers (CT, MRI, ultrasound, triglyceride extraction) or by sampling error (histology). For many studies, particularly those in humans and those where fatty infiltration is not a primary outcome, these methodologies are useful and appropriate. However, for animal studies attempting to delve into the mechanisms that regulate fatty infiltration and its link to muscle dysfunction, precise quantification of the amount and distribution of fatty infiltration is helpful if not necessary. To that end, we have described a novel method to analyze fatty infiltration in mouse muscle that can provide comprehensive qualitative and precise quantitative analysis of fatty infiltrate lipid.

This method begins with a standard decellularization procedure. SDS has been used to decellularize tissues for tissue engineering applications since the early 1990s (reviewed in [41]). A number of groups have noted that in fatty tissues, such as skeletal muscle or adipose tissue, not all lipids are removed by decellularization, and an additional lipid removal step is necessary, either physical dissociation or alcohol extraction [36, 41–44]. Our data suggest that this retained lipid is the large lipid stores of adipocytes trapped in their native anatomical distribution (Fig. 2a–d), rather than lipid membranes or intramyocellular lipid droplets (Fig. 2e). However, the biophysical and biochemical factors that underlie this phenomenon are not well understood [45]. SDS solubilizes lipid membranes by inducing an increase in membrane curvature that is thermodynamically unstable [46]. Thus, the large nonpolar interior of adipocyte lipid combined with the physical constraint of the ECM may act to selectively prevent dissociation from these lipids without additional stimuli. In contrast, the much smaller intramyocellular lipid droplets (of similar size to the mitochondria [47]) may be able to diffuse through and out of the construct. However, more detailed biophysical studies will be necessary to support this hypothesis.

In this work, we demonstrate three ways to use staining of lipid in decellularized muscle to qualitatively or quantitatively describe fatty infiltration. First, ORO staining can be used to qualitatively inspect the 3D pattern of lipid in a decellularized muscle (Fig. 1). In animal studies, fatty infiltration is frequently assessed qualitatively via histology (e.g., [31]). However, typically, only one or a few sections are inspected. This could lead to significant error in comparisons as the pattern of fatty infiltration can be very heterogeneous [33]. This is evident in our data as well. A qualitative histological comparison of 129S1 and BL/6J muscles may come to different conclusions if it is performed on cross-sections taken at the proximal vessel than from the distal half (Fig. 5a). ORO staining of decellularized muscles avoids these sampling errors by providing a comprehensive 3D image of fatty infiltration in the muscle with equal, if not less, effort than required for histological prep.

Second, fluorescent lipid staining combined with confocal microscopy can be used to quantify fatty infiltration at the level of the individual adipocyte (Fig. 3). A few studies have quantified fatty infiltration in animal models with non-invasive imaging such as CT or MRI [25, 26], but the majority has used histology for cell-level metrics (e.g., [32]). This is due in part to the expense of CT and MRI and the limited spatial resolution of these modalities. In this study, we utilized a μCT machine which provided a substantial increase in resolution over conventional CT but still could not distinguish individual adipocytes. Adipocytes could likely be distinguished by nano-CT, but these specialized instruments are not widely available. ORO staining is the gold standard for positively identifying infiltrating adipocytes in muscle sections because eosin-negative areas on H&E could originate from multiple sources [48]. However, it is very difficult to keep lipid from separating and fractionating during sectioning even in fixed tissues at very low temperatures leading to misidentification of lipid areas or volumes (Fig. 4a). For our comparison of adipocyte sizes, we used sequential staining for the lipid droplet membrane protein perilipin, which remains fixed when lipid fractionates, and H&E to doubly confirm adipocyte areas, which was laborious even for the small muscle sub-volumes sampled. By comparison, lipid appears to remain fixed in decellularized muscles and to not coalesce or fractionate (Fig. 4b, c) and can be quantified via a semi-automated image processing procedure.

Additionally, because this technique can be applied through the entire volume of small muscles or to a volume of interest in larger muscles, it can provide additional metrics such as adipocyte volume distributions, adipocyte density (within a muscle or volume), and measures of spatial patterning (e.g., clustering) that cannot be acquired from sampled histological sections. These additional measures may provide data critical to our understanding of fatty infiltration. For example, increased visceral adipocyte volume, more than overall fat mass, is thought to contribute to the development of type 2 diabetes through increased cellular stress [49]. A similar mechanism may explain how intramuscular adipocytes contribute to poor muscle function in pathological conditions. Furthermore, the spatial patterning of fatty infiltration, particularly relative to vessel structures, may shed light on the cellular origin of infiltrating adipocytes as debate continues about whether these adipose progenitors reside in the stromovascular fraction [50]. It is important to note, however, that although the size and general distribution of lipid structures appears to be maintained with decellularization, the absolute distance between structures is likely altered due to contraction of the muscle ECM during decellularization.

Third, should a detailed quantitation of adipocyte metrics not be desired, extraction of ORO-stained lipid and measurement of optical density (OD) can provide a less laborious comparison between muscles/groups (Fig. 6). OD reading of extracted ORO-stained lipid has been used extensively in cell culture studies as a measure of cellular lipid accumulation during adipogenesis (e.g., [51]). In cell culture systems, this technique may suffer from artifacts arising from unbound ORO particulate that can persist even following extensive washing (unpublished observations). However, ORO-stained decellularized muscles can be re-treated with SDS to remove all unbound ORO, resulting in significantly reduced background signal. In fact, ORO staining and extraction performed on SAL-treated TA muscles without infiltrating adipocytes is equivalent to isopropanol controls. In this study, we found a strong correlation between ORO OD and lipid volume measured via confocal microscopy (Fig. 6a), suggesting ORO OD is proportional to the amount of lipid in the construct. However, it is important to note that the relationship is not 1:1, so conclusions about the absolute value of lipid or fold changes in lipid will require the use of a regression line (like that in Fig. 6a) or another standard. Though triglyceride quantification can also be performed on extracted lipid, ORO OD quantification requires fewer reagents and less preparation time. However, should more detailed lipid profiling be desired, extracted lipid could be used for mass spectrometry. This could be useful to compare the lipid profiles between extramyocellular (fatty infiltrate) and intramyocellular lipid stores which are difficult to precisely separate mechanically.

To demonstrate the utility of these three measurement techniques, we have compared GLY-induced fatty infiltration between the two strains of mice: 129S1/Sv1mJ (129S1) and C57BL/6J (BL/6J). Variants of these two strains are commonly used as wildtype and transgenic backgrounds and have been used for a number of investigations into fatty infiltration [37, 39, 40, 52–55]. Thus, it is important to understand whether these strains respond similarly to stimuli for fatty infiltration. Using the decellularization techniques described here, we find substantial differences between strains via qualitative inspection of the ORO-stained lipid (Fig. 5a), quantitative analysis of lipid droplet metrics via confocal microscopy (Fig. 5b–e) and quantitation of extracted ORO-stained lipid (Fig. 6b).

GLY injections into the 129S1 muscle induced over five times the amount of fatty infiltration as in the BL/6J muscle (Fig. 5b). We confirmed a similar degree of regeneration (a surrogate measure of GLY penetration) between the strains with histological analysis of centralized nuclei, suggesting that our results are not due to differences in injection efficiencies. Furthermore, a similar but less dramatic (~0.5–2-fold) difference was also found in another study in the thigh and popliteal regions of these strains following GLY injections [40], suggesting an intrinsic difference between strains in susceptibility to GLY-induced fatty infiltration. It is tempting then to suggest that the 129S1 mice are a better model for fatty infiltration. However, the distribution of lipid also differs between these strains, even when fatty infiltrate volume is relatively similar (compare Fig. 5a top panel with Fig. 2a) suggesting that mechanisms that regulate the differentiation and migration of fatty infiltrate progenitor cells, and the progenitor cells themselves, could differ between these strains. Whether these differences are limited to the response to GLY injection or are a systemic difference in the sensitivity of a progenitor cell to multiple drivers of fatty infiltration remains to be evaluated. It has been suggested that the fatty infiltrate in 129S1 muscle is brown fat [40], which shares a developmental lineage with satellite cells, while the fatty infiltrate in BL/6J is not brown and may arise from progenitors in the stromovascular fraction [53, 54]. We found no evidence of multilocular lipid typical of brown fat in either strain by confocal imaging or histology, but that does not rule out phenotypic differences between these two strains as beige fat can resemble brown fat metabolically and white fat morphologically [56].

Moving both mechanistic studies and disease models forward, mouse background should be considered carefully. Our data suggest that the background strain of the mouse may contribute to its susceptibility to pathology under the same experimental drivers, much like human genetics may contribute to whether an obese individual is metabolically normal or abnormal. As mouse models of disease struggle to recapitulate features of human disease, it may be worthwhile to consider whether the model may be on a background resistant to that feature and which background(s) best represent the human disease process.

Though this work has focused on utilizing decellularization-based imaging methodology to quantify fatty infiltration between wildtype strains, application of these techniques to disease models such as the mdx mouse (the model for Duchenne muscular dystrophy) is a rich area for future studies. However, moving forward, it is important to note that these models exhibit additional features of muscle pathology that could confound application of this methodology. Increased ECM content (fibrosis) could potentially impede the removal of cellular materials, increasing required SDS incubation times. However, though limited to a single sample, we encountered no difficulties using the methodology described here to decellularize and stain a fibrotic human muscle biopsy (Additional file 2: Figure S1). Significant atrophy present in some disease models may also require the analysis of both absolute and normalized metrics of fatty infiltration to determine whether the absolute amount of lipid or percentage of lipid relative to muscle volume are the most meaningful outcome measures.

While decellularization-based imaging methodology offers several advantages over current techniques for analyzing fatty infiltration, it also has several limitations. The most significant limitation is that cellular measurements cannot be made following decelluarization. Functional measures on intact muscles (e.g., active physiology or mitochondrial respiration) can be run prior to decellularization, but morphological measures (e.g., fiber size, capillary density, or protein immunostaining) cannot be run in conjunction with decellularization the way they can with histological quantification of fatty infiltration. Quantification of adipocyte-level metrics is also limited to thin muscles or surface regions of interest by the penetration depth of the confocal microscope. Thus, for larger muscles, this analysis will suffer from the same sampling issues that histology typically does. Furthermore, decellularization cannot be used for longitudinal studies the way non-invasive measurement techniques such as CT or MRI can. Thus, it will be important for each individual study to select methodology based on the experimental questions being asked and the level of data desired. Some of the potential applications to benefit from this methodology include studies where fatty infiltration is a primary outcome measure, studies whose other outcome measures are functional measures in intact muscle, studies whose other outcome measures are in a different muscle (i.e., multiple muscles affected by a treatment), or studies in which tissue availability is not a limitation.

## Conclusions

We have developed a novel method for qualitatively and quantitatively analyzing fatty infiltration in muscle by staining and imaging lipid retained in decellularized constructs. This methodology can provide a comprehensive image of the 3D pattern of fatty infiltration in muscle and a quick quantification via ORO staining, without the need for access to a cryostat, CT, or MRI. If more detailed measures are desired, it can also provide quantification of adipocyte numbers, sizes, and distribution patterns via confocal imaging of fluorescent-stained lipid. These measures provide researchers a way to precisely measure fatty infiltration at the level of the adipocyte throughout a small muscle volume without extensive serial sectioning or very high-powered specialized CT machines.

## Additional files

Additional file 1: Figure S2. Illustration of the image registration procedure used to analyze fluorescent regions of interest (ROIs) in confocal stacks with ImageJ and Matlab. ImageJ menu navigation commands are listed in orange throughout. (A) A representative slice in a confocal stack. (B) Application of the Intermodes thresholding algorithm colors thresholded areas red in the preview screen. (C) following thresholding, the slice is converted to a binary image. Application of the Watershed algorithm segments the thresholded area based on circularity. (D) The Analyze Particles algorithm registers segmented areas into numbered ROIs. (E) Thresholding, segmentation and ROI registration are performed simultaneously for the entire confocal stack. Viewing all ROIs simultaneously provides a compressed view of the entire stack registration. Any areas of weak signal that were excluded from the thresholding can be registered manually using the ImageJ drawing tools and ROI manager (e.g., perfect circles in panel E). (F) An individual lipid droplet may be registered as an ROI in multiple slices. To prevent multiple registration, only the largest area ROI for each droplet was retained and then converted to a volume as described in the Methods. This refinement was performed in Matlab using the m-file Refine.m provided with this publication. This is necessary because out-of-plane fluorescent artifacts cause a lipid droplet to appear to be an elongated ellipsoid along the *Z*-axis if volume is computed by summing ROIs in each slice. (TIF 12245 kb)
Additional file 2: Figure S1. Decellularization of different muscles from five anatomical locations in the mouse and a human biopsy. Images of decellularized and ORO-stained muscles are shown below the corresponding intact muscle image and denoted by primed letters. (a) Tibialis anterior, (b) 5th toe extensor digitorum longus, (c) muscle groups of the distal hindlimb with maintained skeletal attachments, (d) supraspinatus, (e) infraspinatus, (f) muscles of the rotator cuff with maintained scapular attachments, (g) biceps lateral head, (h) brachioradialis, (i) muscle groups of the distal forelimb with maintained skeletal attachments, (j) portion of the rectus abdominis, (k) diaphragm, (l) biopsy of a human gastrocnemius muscle. (TIF 8629 kb)
Additional file 3: Supplemental Methods. (DOCX 11 kb)

## Abbreviations

129S1: 129S1/Sv1mJ mouse strain
BL/6J: C57BL/6J mouse strin
CT: Computed tomography
ECM: Extracellular matrix
EDL: Extensor digitorum longus muscle
Gastroc: Gastrocnemius muscle
GLY: Glycerol
H&E: Hematoxylin & eosin
MRI: Magnetic resonance imaging
OD: Optical density
ORO: Oil Red O
ROI: Region of interest
SAL: Saline
SDS: Sodium dodecyl sulfate
TA: Tibialis anterior muscle
μCT: Micro-computed tomography
