# Supplementary figures and images for: A novel method for the quantification of fatty infiltration in skeletal muscle
Source: Skelet Muscle. 2017 Jan 10;7:1. doi: 10.1186/s13395-016-0118-2 (PMC5223468; doi:10.1186/s13395-016-0118-2)

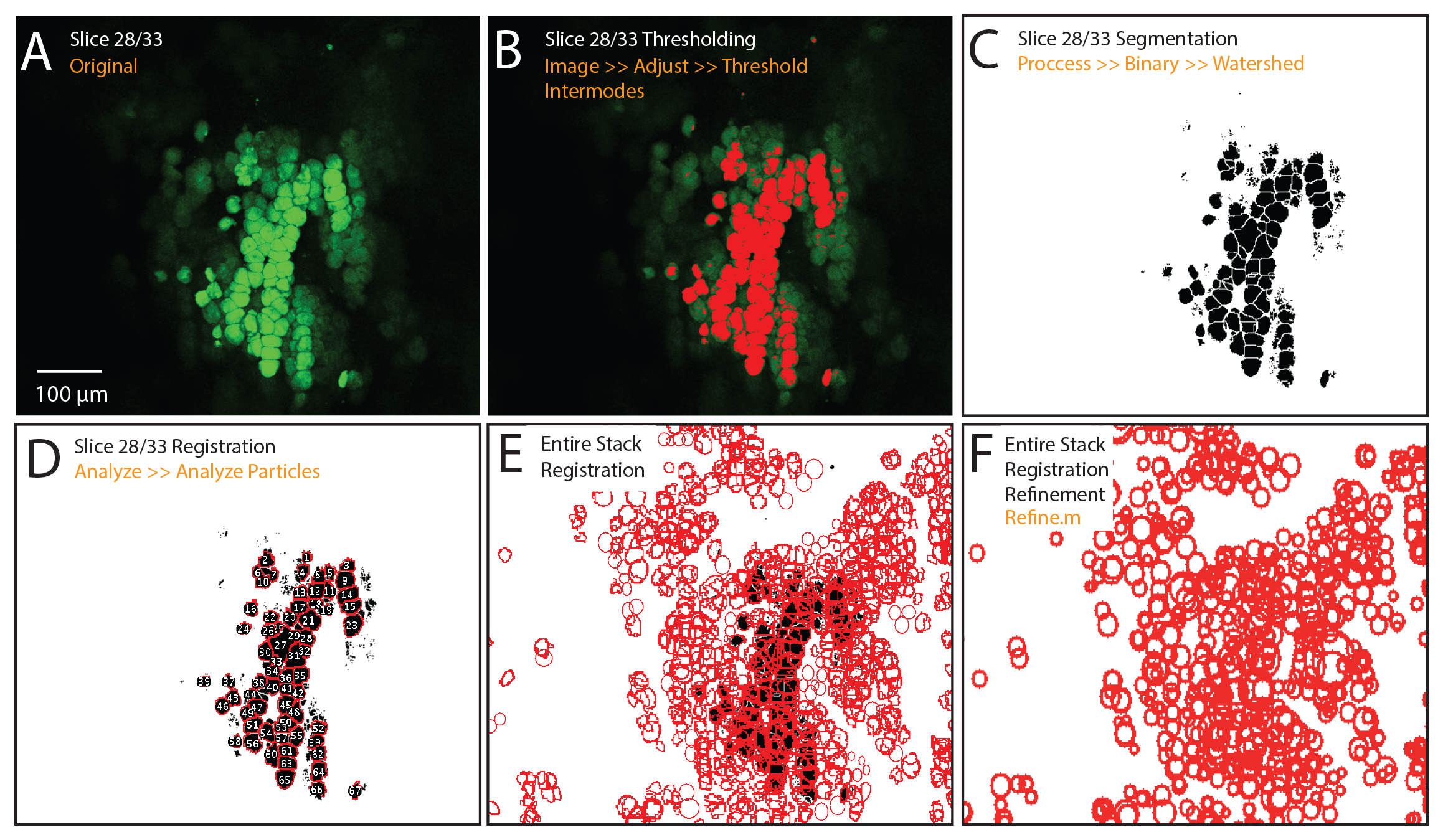

Supplement: Additional file 1: Figure S2. — Illustration of the image registration procedure used to analyze fluorescent regions of interest (ROIs) in confocal stacks with ImageJ and Matlab. ImageJ menu navigation commands are listed in orange throughout. (A) A representative slice in a confocal stack. (B) Application of the Intermodes thresholding algorithm colors thresholded areas red in the preview screen. (C) following thresholding, the slice is converted to a binary image. Application of the Watershed algorithm segments the thresholded area based on circularity. (D) The Analyze Particles algorithm registers segmented areas into numbered ROIs. (E) Thresholding, segmentation and ROI registration are performed simultaneously for the entire confocal stack. Viewing all ROIs simultaneously provides a compressed view of the entire stack registration. Any areas of weak signal that were excluded from the thresholding can be registered manually using the ImageJ drawing tools and ROI manager (e.g., perfect circles in panel E). (F) An individual lipid droplet may be registered as an ROI in multiple slices. To prevent multiple registration, only the largest area ROI for each droplet was retained and then converted to a volume as described in the Methods. This refinement was performed in Matlab using the m-file Refine.m provided with this publication. This is necessary because out-of-plane fluorescent artifacts cause a lipid droplet to appear to be an elongated ellipsoid along the Z-axis if volume is computed by summing ROIs in each slice. (TIF 12245 kb) [file 13395_2016_118_MOESM1_ESM.tif]

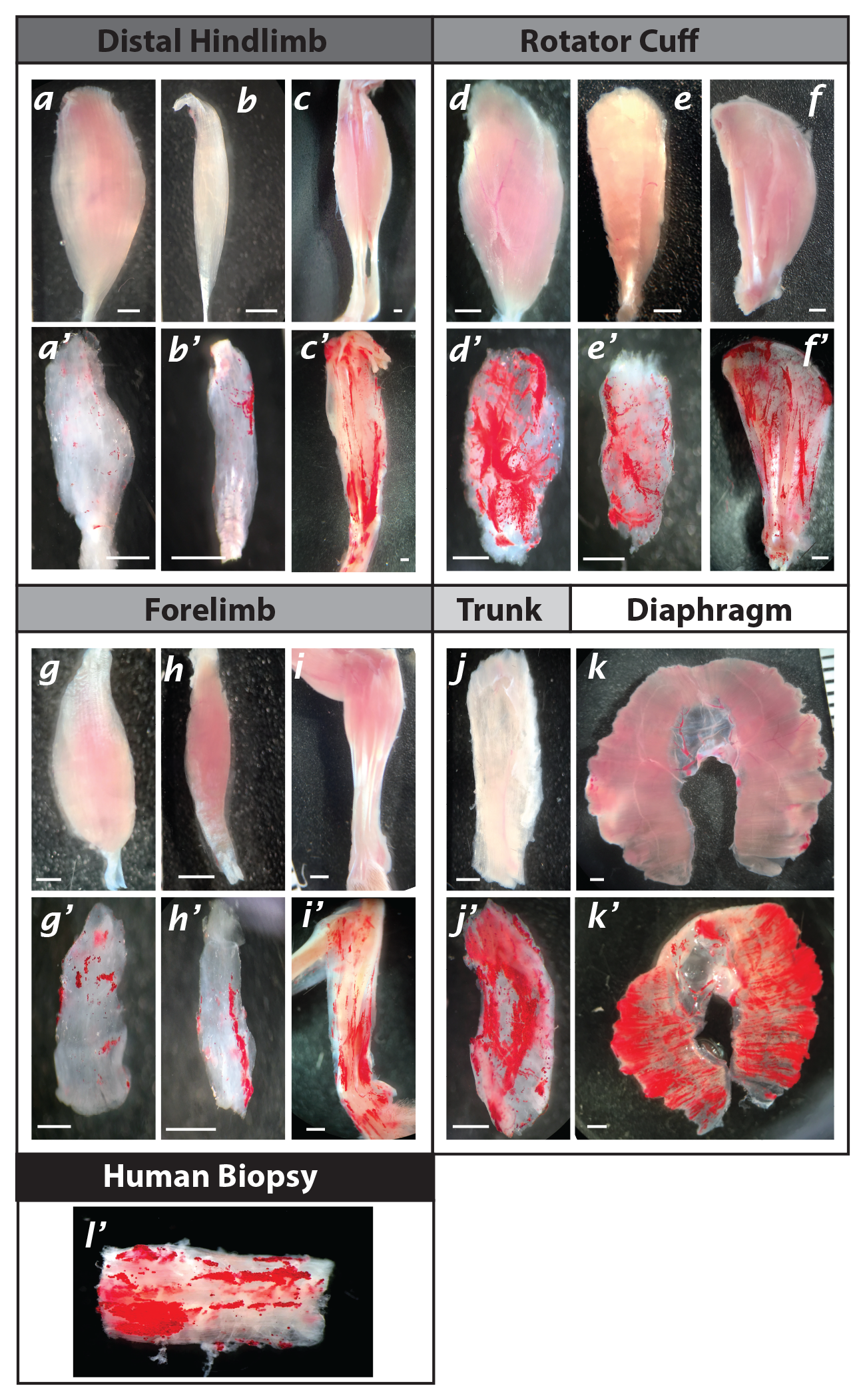

Supplement: Additional file 2: Figure S1. — Decellularization of different muscles from five anatomical locations in the mouse and a human biopsy. Images of decellularized and ORO-stained muscles are shown below the corresponding intact muscle image and denoted by primed letters. (a) Tibialis anterior, (b) 5th toe extensor digitorum longus, (c) muscle groups of the distal hindlimb with maintained skeletal attachments, (d) supraspinatus, (e) infraspinatus, (f) muscles of the rotator cuff with maintained scapular attachments, (g) biceps lateral head, (h) brachioradialis, (i) muscle groups of the distal forelimb with maintained skeletal attachments, (j) portion of the rectus abdominis, (k) diaphragm, (l) biopsy of a human gastrocnemius muscle. (TIF 8629 kb) [file 13395_2016_118_MOESM2_ESM.tif]
