# Supplementary material for: A novel method for the quantification of fatty infiltration in skeletal muscle
Source: Skelet Muscle. 2017 Jan 10;7:1. doi: 10.1186/s13395-016-0118-2 (PMC5223468; doi:10.1186/s13395-016-0118-2)
Supplement: Additional file 3: — Supplemental Methods. (DOCX 11 kb) [file 13395_2016_118_MOESM3_ESM.docx]

SUPPLEMENTAL METHODS

*Decellularization of Mouse and Human Muscle for Supplemental Figure 1*

Mouse muscles were obtained from a single C57BL/6J male mouse at 18 months of age subjected to no experimental manipulation. The human muscle biopsy was obtained from the Gastrocnemius muscle of an individual undergoing below-knee amputation. This biopsy was obtained with written consent from the subject under the Institutional Review Board of Washington University in St. Louis.

*Matlab Code for ROI Refinement*

function mat=refine(original)

% This function eliminates repeat measures of the same lipid droplet in thresholded structures from ImageJ

% MAT = [NUMBER AREA X Y MAJOR MINOR ANGLE SLICE]

mat=original;

j=1;

while j<length(mat)

% Find all ROI centroids within a set tolerance distance from the jth ROI

ind=find(mat(j,3)*0.99<mat(:,3) & mat(:,3)<mat(j,3)*1.01);

mat_tmp=mat(ind,:);

jnd=find(mat(j,4)*0.99<mat_tmp(:,4) & mat_tmp(:,4)<mat(j,4)*1.01);

mat_tmp_tmp=mat_tmp(jnd,:);

% Select the largest ROI from the set with overlapping ROI centroids

[mx knd]=max(mat_tmp_tmp(:,2));

% Remove the others from the matrix

jnd(knd)=[];

mat(ind(jnd),:)=[];

clear ind jnd knd

j=j+1;

end
